# Clinical Impact of Ultrafast Cranial MRI Implementation in Children Under Six Years of Age

**DOI:** 10.3390/jcm15031242

**Published:** 2026-02-04

**Authors:** Rastislav Pjontek, Hani Ridwan, Benedikt Kremer, Michael Veldeman, Dimah Hasan, Martin Häusler, Martin Wiesmann, Hans Clusmann, Hussam Hamou

**Affiliations:** 1 Department of Neurosurgery, University Hospital RWTH Aachen, 52074 Aachen, Germanyhhamou@ukaachen.de (H.H.); 2 Department of Diagnostic and Interventional Neuroradiology, University Hospital RWTH Aachen, 52074 Aachen, Germany; 3 ^3^ Division of Neuropediatrics and Social Pediatrics, Department of Pediatrics, University Hospital RWTH Aachen, 52074 Aachen, Germany

**Keywords:** ultrafast cranial MRI, ultrafast brain MRI, ultra-rapid MRI, rapid sequence MRI, quick brain MRI, hydrocephalus, ventriculoperitoneal shunt, pediatric TBI, radiation reduction

## Abstract

**Background**: Young children requiring neurosurgical care frequently undergo repeated neuroimaging. Whereas CT involves exposure to ionizing radiation, conventional MRI is time-consuming and often necessitates sedation in non-cooperative children. To address these limitations, ultrafast cranial MRI (UF-MRI) based on T2-HASTE sequences was implemented at our institution in 2019 for selected indications. The aim of this study was to evaluate the real-world implementation of UF-MRI in children younger than six years of age. **Methods**: We retrospectively analyzed cranial MRI examinations consisting exclusively of ultrafast sequences performed between July 2019 and December 2024 in children younger than six years. Clinical settings, diagnostic adequacy, immediate consequences for patient management, and the impact on MRI and CT utilization were systematically assessed. **Results**: A total of 404 UF-MRI examinations were performed in 198 inpatients and outpatients (mean age: 2 years 2 months) without the need for dedicated anesthesia team support solely for imaging. Only one examination (0.2%) required same-day repetition after mild oral sedation. In 20 patients (5.0%), UF-MRI was supplemented by conventional MRI under anesthesia, most commonly for preoperative planning. Immediate clinical consequences included no change in management in 54.5% of examinations, early follow-up in 22.8%, shunt valve adjustment in 11.6%, neurosurgical intervention in 7.7%, and other measures in 5.0%. UF-MRI accounted for 24.5% of all cranial MRI examinations in this age group and was associated with a 41% reduction in CT utilization compared with the corresponding period prior to UF-MRI implementation. **Conclusions**: In routine clinical practice, UF-MRI provides rapid, clinically sufficient neuroimaging in young children without the need for sedation or exposure to ionizing radiation. Its implementation significantly streamlines imaging workflows, optimizes resources utilization, reduces the need for CT, and supports timely clinical decision-making, underscoring its value as a complementary imaging modality in pediatric neuroimaging.

## 1. Introduction

Children with neurosurgical conditions frequently require repeated neuroimaging for diagnosis, treatment planning and follow-up. While conventional cranial MRI (cMRI) represents the diagnostic gold standard, long acquisition times and susceptibility to motion artifacts often necessitate sedation or general anesthesia in infants and young children. These procedures are associated with considerable logistical, healthcare staff-related, financial, and organizational burden, and carry inherent medical risks [1].

Cranial computed tomography (cCT) and ultrasound represent established alternative imaging modalities to MRI. Ultrasound is examiner-dependent and restricted to the period before fontanelle closure, generally providing reliable brain imaging only during the first six months of life [2], while cCT offers rapid image acquisition but at the cost of ionizing radiation. Large cohort studies have demonstrated a dose-dependent association between pediatric CT exposure and the later development of hematological malignancies and brain tumors [3,4,5,6,7]. In children with shunted hydrocephalus, with an average neuroimaging frequency of 2.1 studies per year, repeated CT surveillance throughout childhood has been estimated to result in one excess lifetime fatal cancer per 97 patients with standard cCT protocols, or per 230 patients with low-dose protocols [8]. These findings underscore the importance of minimizing radiation exposure and prioritizing radiation-free alternatives whenever diagnostically sufficient. To address these limitations, ultrafast (also referred to as ultra-rapid or quick brain) cranial MR sequences have been developed.

First introduced in the early 2000s [9], these protocols, typically based on heavily T2-weighted sequencies, enable acquisition times typically of less than two minutes without need for sedation. They show high tolerance to patient motion and maintain diagnostic utility even in non-cooperative children, albeit at the cost of reduced contrast and spatial resolution [10]. Their earliest and most common application has been the evaluation of cerebrospinal fluid (CSF) spaces in children with or without shunts [11,12,13]. Adoption has been widespread: in a North American survey, 79% of 56 pediatric institutions had implemented rapid-sequence MRI for ventricular size assessment, and 64% reported routine use [14]. Over time, indications have expanded from shunted hydrocephalus to ventriculomegaly, macrocephaly, intracranial cysts [15], abusive head trauma [16,17], emergency imaging for persistent/recurrent headache [18] and traumatic brain injury (TBI) [19,20,21,22,23,24,25].

Beyond diagnostic performance, UF-MRI offers substantial advantages in terms of workflow efficiency, resource utilization, and patient safety. In addition to markedly reduced scanning times [26], UF-MRI has been associated with significant cost savings, primarily due to the reduced need for anesthesia and related resources [10,27].

Despite these advantages, most prior studies have focused on technical feasibility or qualitative comparisons with conventional MRI [26] or CT [20,21,22], whereas systematic evaluations of UF-MRI implementation in routine clinical practice and its impact on clinical decision-making and cross-sectional imaging utilization remain scarce. To address this gap, the aim of this study was to evaluate the real-world implementation of UF-MRI in children younger than six years at a tertiary care center with particular focus on clinical indications, diagnostic yield, consequences for patient management, examination logistics, and its role in reducing CT utilization.

## 2. Materials and Methods

### 2.1. Ethics Approval

The study was approved by the local institutional ethics committee and conducted in accordance with the Declaration of Helsinki. Due to the retrospective analysis of patient data, informed consent was waived (local registration number: EK 25-376).

### 2.2. Study Population

This monocentric retrospective analysis included all UF-MRI examinations performed between 1 July 2019 and 31 December 2024, in pediatric patients younger than 6 years at the time of imaging. An additional prerequisite was the availability of clinical data regarding patient history, indication for imaging, and follow-up after the examination.

Exclusion criteria were ultrafast sequences performed solely as part of a conventional cranial MRI protocol, hybrid protocols combining ultrafast with additional conventional sequences, or ultrafast sequences acquired for spinal imaging.

### 2.3. UF-MRI Acquisition Workflow

The child and one parent (or caregiver) are escorted from the waiting area, metallic objects are checked for and removed, and the child is positioned on the examination table. One parent is allowed to lie on the MRI table alongside the child, wearing hearing protection, to provide physical contact for reassurance. The table is then advanced into the MRI scanner, the sequences are aligned to the localizer, loaded, and acquired. Sequence quality is assessed rapidly during the examination, and in cases of motion artifacts, either individual sequences or all three planes may be repeated. Alternatively, in clearly restless children, the sequences may be preloaded two or three times at the outset using a “shotgun” approach, increasing the likelihood of obtaining at least one motion-free set, thus supporting reliable interpretation and diagnostic confidence. To minimize patient motion during the examination, several further measures were employed when necessary: the “feed and wrap” technique in neonates, allowing a parent to lie on the MRI table while gently stabilizing the child’s head, or oral administration of an antihistamine syrup. Critically ill inpatients (e.g., after status epilepticus, traumatic brain injury, or surgery) were accompanied by anesthesia or intensive care staff when clinically indicated.

### 2.4. MRI Technique and Sequence Parameters

MRI examinations were performed using a 1.5 Tesla scanner (MAGNETOM Aera, Siemens Healthcare, Erlangen, Germany) equipped with a 20-channel head coil. The imaging protocol consisted of T2-weighted HASTE (Half-Fourier Acquisition Single-Shot Turbo Spin-Echo) sequences acquired in axial, coronal, and sagittal orientations.

The acquisition times for the sequences were as follows: axial 22 s, sagittal 23 s, and coronal 27 s. An additional 10 s were required for the localizer scan used for planning.

The technical parameters for the T2-HASTE sequences were as follows:

- Slice thickness (SD): 5.00 mm;
- Slice spacing (SA): 6.50 mm;
- Field of view (FOV): 150.0 mm;
- Repetition time (TR): 1000 ms;
- Echo time (TE): 78 ms;
- Number of signal averages (NSA): 1;
- Turbo spin echo factor (TSE): 82.

### 2.5. Data Collection and Analysis

Clinical, radiological, and procedural data were retrospectively extracted from the hospital and radiology information system, including medical records, surgical reports, radiological findings, daily clinical notes and MRI images.

Collected variables included patient demographics, primary diagnosis, clinical setting, imaging indications, UF-MRI acquisition parameters (number of acquired and repeated sequences, total scan time), imaging findings, the presence of motion artifacts, the need for conventional MRI, and immediate clinical consequences following UF-MRI.

Imaging findings were categorized as normal, newly diagnosed pathology, stable findings, progression or regression of known disease. Motion artifacts were classified based on visual assessment of all acquired sequences: “absent” if no motion artifacts were visible; “mild” if motion artifacts were present but did not impair diagnostic confidence; and “pronounced” if motion artifacts affected at least one slice in one or more sequences but the overall protocol still allowed the clinical question to be answered. An examination was considered diagnostically inadequate if it had to be repeated at another time, because the clinical question could not be reliably addressed. Based on a joint review of the radiology report and subsequent clinical management, an examination was classified as clinically sufficient if the UF-MRI findings were adequate to guide clinical decision-making without the need for additional cross-sectional imaging.

Immediate clinical consequences were defined as diagnostic or therapeutic actions directly attributable to UF-MRI findings and initiated on the same day or during the same hospitalization. These included no change in management, early follow-up imaging, shunt valve reprogramming, surgical intervention, or other targeted clinical measures.

Additionally, the number of conventional MRI and cranial CT scans performed in the same pediatric age group was extracted from the radiology information system for two five-and-a-half-year periods, pre-implementation (2014–mid-2019) and post-implementation (mid-2019–2024) of UF-MRI, in order to evaluate a potential reduction in CT utilization. Descriptive statistical analyses were performed using appropriate software tools.

## 3. Results

### 3.1. Characteristics of the Study Cohort, Indications and Clinical Context

Characteristics of the study cohort are summarized in Table 1. The final cohort comprised 404 UF-MRI examinations performed in 198 children (85 females and 113 males). The mean age at imaging was 2 years and 2 months (range: 1 day to 5 years 11 months; standard deviation: 560 days). Hydrocephalus was the most frequent underlying condition (n = 210; 52.0%), followed by traumatic brain injury (n = 59; 14.6%), subdural hematomas or hygromas (n = 49; 12.1%), and intracranial cysts (n = 14; 3.5%). In 72 examinations, other diagnoses were present.

The indications for UF-MRI reflected the composition of the study cohort. In 228 cases (56.4%), imaging was performed to assess ventricular size, followed by evaluation of subdural hematoma dynamics (n = 92; 22.8%). Forty-seven examinations (11.6%) were conducted after head trauma to detect intracranial sequelae. Beyond established indications, UF-MRI was also used for orientation in suspected epileptogenic lesions in patients with first epileptic seizures (n = 32; 7.9%), for the evaluation of headache and vomiting with suspected space-occupying lesions (n = 15; 3.7%), and for macrocephaly or developmental delay (n = 13; 3.2%).

Acutely symptomatic patients accounted for 175 examinations (43.3%), follow-up of known pathologies for 209 (51.7%), and elective baseline imaging—for example, in developmental disorders or preoperative work-up for craniosynostosis correction—for 20 cases (5.0%). UF-MRI was performed in 189 outpatients (46.8%) and 215 inpatients (53.2%).

No UF-MRI examination required dedicated anesthesia team support and general anesthesia solely for the imaging procedure.

### 3.2. Examination Metrics and Motion Artifacts

In this cohort, a total of 1381 HASTE sequences were acquired, corresponding to a mean of 3.44 sequences per examination (SD 0.84). Axial sequences were most frequently performed. In 144 instances, individual sequences were repeated twice, and in 31 instances, three times. In five examinations, a total of seven additional axial TrueFISP (Trufi) sequences were acquired.

In 297 examinations (73.5%), the total scan time was less than 2 min, while only 11 (2.7%) examinations exceeded 5 min—primarily in patients who moved during the scan, necessitating sequence repetition and, in some cases, patient repositioning.

No motion artifacts were observed in 130 examinations (32.2%), while 112 (27.7%) showed mild and 162 (40.1%) pronounced artifacts. Motion artifacts were strongly age-dependent, with image quality improving with increasing age. Pronounced artifacts were most frequent in infants aged 0–12 months (56.0%), decreased in children aged 1–3 years (41.9%), and were the least common in those aged 4–6 years (21.1%), in whom over half of examinations were artifact-free (50.5%).

Only one case was considered diagnostically inadequate and required same-day repetition of UF-MRI following mild oral sedation. Motion artifacts did not necessitate additional conventional MRI when UF-MRI satisfied the clinical question.

### 3.3. Findings and Immediate Clinical Consequences

Image findings on UF-MRI were heterogeneous and, in some cases, comprised more than one relevant morphological change within a single examination (e.g., decreasing ventricular size accompanied by increasing subdural hygromas).

A normal imaging result was observed in 75 examinations (16.8%). A newly detected pathological finding representing an initial diagnosis was identified in 45 cases (10.1%), while 121 examinations (27.1%) demonstrated unchanged findings compared with prior imaging. Changes in ventricular size were common: ventricular enlargement was noted in 52 examinations (11.6%), whereas a reduction in ventricular width was observed in 56 cases (12.5%). Progression of the underlying pathology was documented in 16 examinations (3.6%), while radiological regression of previously known findings was seen in 82 cases (18.3%).

**Figure 1:**
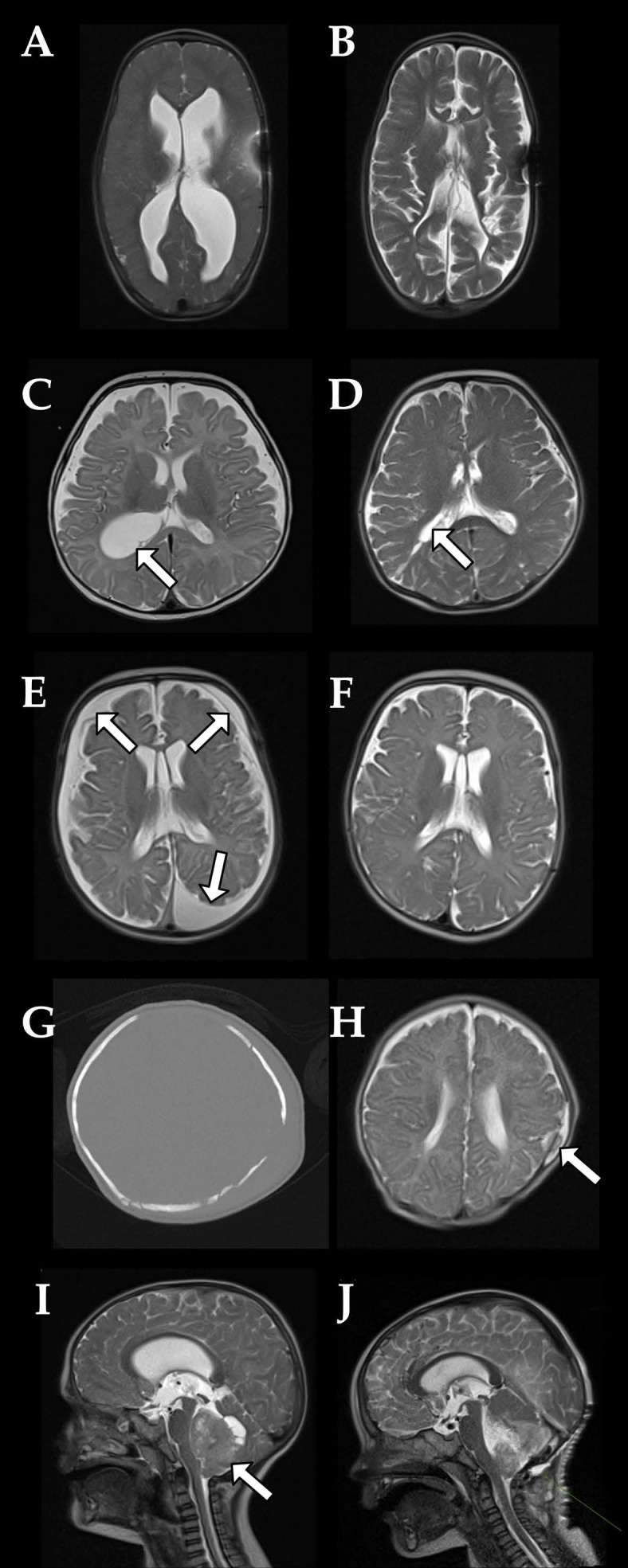
Illustrative clinical applications of ultrafast MRI in pediatric neurosurgical patients. **Case 1:** Posthemorrhagic hydrocephalus after intraventricular hemorrhage (IVH) with parenchymal involvement in one of the preterm triplets. Following ventriculoperitoneal (VP) shunt placement, serial follow-up was performed using UF-MRI. (**A**): shunt dysfunction with ventricular enlargement. (**B**): postoperative control after operative shunt revision and valve replacement. **Case 2:** A 4-month-old girl presenting with rapid head circumference growth and a setting-sun sign. Conventional MRI including T2-TSE demonstrated an intraventricular cyst at the right trigone (**C**, arrow). The lesion was treated by endoscopic cyst fenestration via a right parietal approach. One-year postoperative follow-up using UF-MRI (**D**) demonstrates cyst resolution (arrow) and normalization of ventricular size. **Case 3:** A 2-month-old boy with suspected abusive head trauma. UF-MRI revealed multiphase bilateral subdural hematomas (**E**, arrows). Following surgical evacuation of the hematomas, follow-up imaging (**F**) demonstrated marked regression of the findings. **Case 4:** A 2-month-old girl with traumatic encephalocele due to dural tear and growing skull fracture. The infant’s mother fell on asphalt while carrying her in a baby sling. Initial CT revealed multiple, partially displaced skull fractures (**G**). Follow-up UF-MRI (**H**) demonstrated subsequent encephalocele formation (arrow) secondary to a dural tear with progressive widening of the fracture. Surgical repair of the dura and calvarial defect was performed. **Case 5:** A 2.5-year-old boy presenting with subacute ataxia, tendency to fall to the left, and new-onset vomiting. UF-MRI performed on the day of admission to the pediatric emergency department revealed a posterior fossa mass (**I**, arrow). Following preoperative planning MRI, surgical resection was performed. Early postoperative orientation MRI (**J**) obtained within hours after resection demonstrates an expected postoperative appearance with a visible resection cavity and regression of ventricular enlargement.

**Figure 2:**
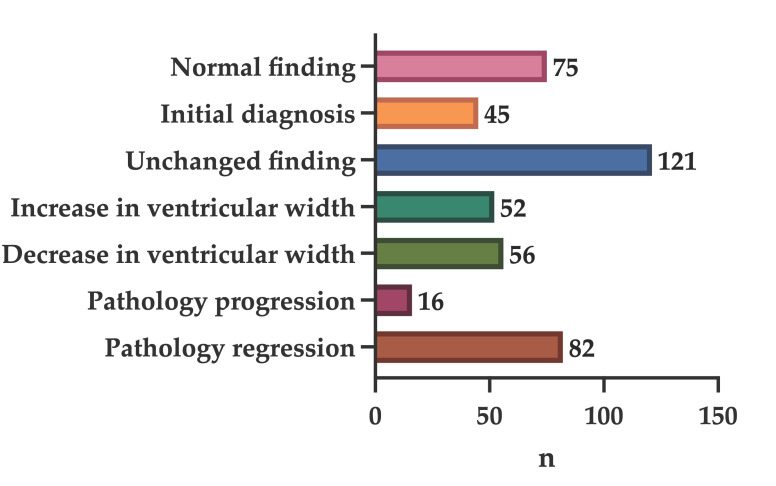
Distribution of imaging findings on ultrafast MRI examinations.

Following this overview, Figure 1 provides representative illustrative cases demonstrating the spectrum of UF-MRI findings across different clinical scenarios, while Figure 2 summarizes the distribution of imaging findings and their frequencies in graphical form.

A particular focus of the analysis was the precise assessment of the immediate clinical consequences derived from UF-MRI findings. In 220 examinations (54.5%), the imaging did not result in any change in subsequent clinical management, either because the findings were normal in first-time examinations or remained unchanged—most commonly in patients with hydrocephalus with or without a shunt, showing stable ventricular size.

In 92 cases (22.8%), no active intervention was required; however, the imaging findings prompted early follow-up, typically within three months, usually with UF-MRI or, in cases with an open fontanelle, with cranial sonography.

In 47 cases (11.6%), deliberate shunt valve pressure reprogramming was performed. Of these, 30 cases (7.4%) involved lowering the valve pressure setting to increase CSF drainage, while in 17 cases (4.2%) the pressure was increased—for example, in patients with slit ventricles or hygromas as a sign of overdrainage.

In 31 cases (7.7%), UF-MRI findings led to the decision to proceed with surgery. Surgical procedures most frequently involved shunt-related interventions, including shunt implantation (ventriculoperitoneal or ventriculoatrial), operative shunt exploration with valve revision, ventricular catheter replacement, placement of additional ventricular catheter and re-internalization of externalized shunts. Other neurosurgical procedures comprised tumor resection (medulloblastoma), third ventriculocisternostomy, evacuation of subdural hematomas, duraplasty after traumatic dural tear, and cyst fenestration. In 20 cases (5.0%), other clinical measures were undertaken based on UF-MRI results including clamping or removal of external ventricular drains (EVD) or subdural drains, adjustment of EVD height, lumbar puncture, or puncture of a Rickham reservoir. In a single case, rapid tumor progression led to the transition to palliative care.

**Figure 3:**
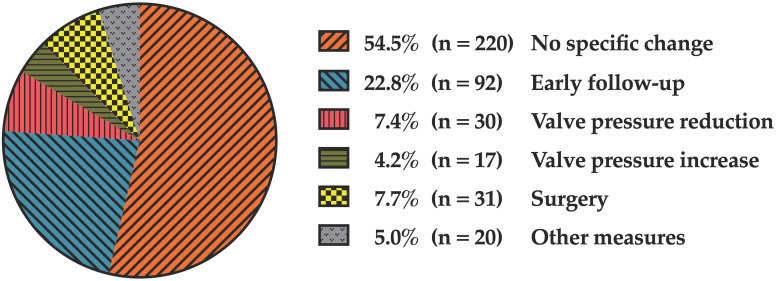
Immediate clinical consequences of UF-MRI (multiple consequences possible).

A detailed overview of these management outcomes is illustrated in Figure 3.

The distribution of clinical consequences across diagnostic categories is summarized in Supplementary Table S1. In both hydrocephalus after IVH and hydrocephalus of other origin, UF-MRI frequently led to active management changes (over 58% in both groups). Valve pressure adjustments were significantly more common in post-hemorrhagic hydrocephalus (29.0%) than in hydrocephalus of other origin (13.1%; Fisher’s exact test, *p* = 0.0055), whereas rates of surgical intervention were comparable (11.8% vs. 11.5%). In patients with subdural hematomas, early follow-up was the most frequent consequence (53.1%). In trauma cases, UF-MRI prompted surgical intervention in one patient, while approximately one quarter of examinations (those with intracranial trauma sequelae) resulted in early follow-up imaging. Examinations performed for intracranial cysts most often resulted in no change in management (85.7%).

In 20 patients, UF-MRI was supplemented by conventional MRI sequences under anesthesia—most commonly for preoperative planning (e.g., acquisition of 3D navigation sequences, detailed tumor work-up prior to resection, or depiction of cyst walls and their relation to the ventricular system for fenestration planning). The remaining cases were epilepsy patients undergoing dedicated high-resolution imaging to localize an epileptogenic focus. In none of the cases did the findings of the conventional MRI contradict those of the UF-MRI.

### 3.4. UF-MRI in the Context of Cross-Sectional Imaging in Children up to Six Years of Age

During the 5.5-year period following the implementation of UF-MRI (1 July 2019 to 31 December 2024), a total of 1650 neurocranial MRI examinations were performed in children younger than six years at our institution. Of these, 404 examinations (24.5%) consisted exclusively of ultrafast MRI protocols. Compared with the preceding 5.5-year period prior to UF-MRI implementation (1 January 2014 to 30 June 2019), during which 1175 neurocranial MRI examinations were performed in the same age group, this represents an absolute increase of 475 examinations, corresponding to a 40.4% rise in MRI utilization.

In 84 cases (5.1% of all MRI examinations in this age group), UF-MRI was supplemented with additional conventional sequences such as T2*, T2-FLAIR, or diffusion-weighted imaging (DWI)—most often in emergency settings such as trauma, particularly when patients were sufficiently cooperative to tolerate the longer acquisition times.

**Figure 4:**
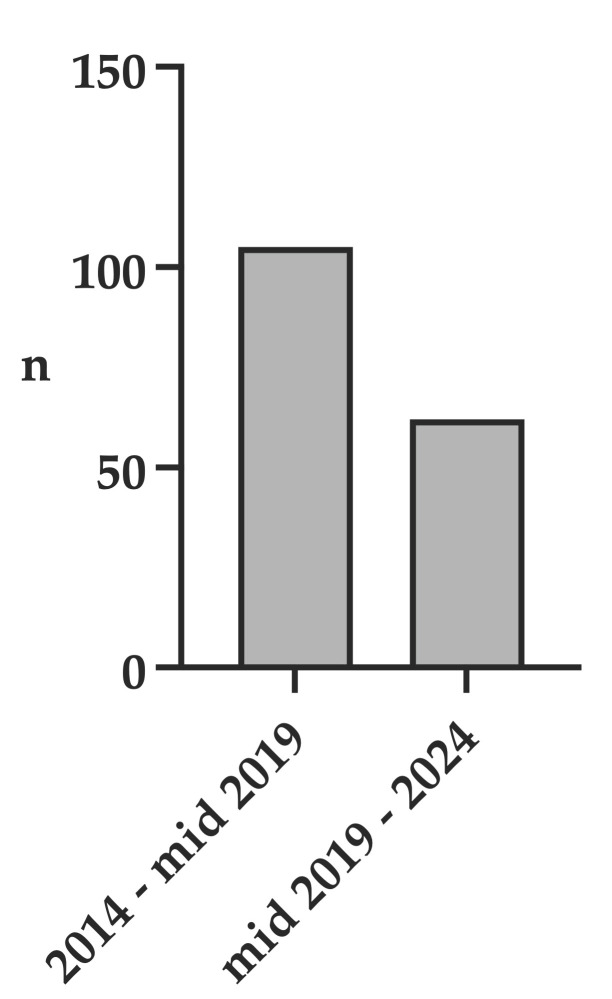
Number of cranial CT examinations in children < 6 Years before and after introduction of ultrafast MRI.

In this five-and-a-half-year period prior to UF-MRI implementation (2014–mid 2019), 105 cranial CT (cCT) examinations were performed in children younger than six years. In the corresponding five-and-a-half-year period following UF-MRI implementation (mid 2019–2024), the number of cCT examinations decreased to 62, representing a 41.0% reduction (Figure 4).

## 4. Discussion

This study represents one of the largest, and potentially the largest, analyses of ultrafast cranial MRI in children younger than six years. Beyond demonstrating feasibility, it highlights the clinical impact of UF-MRI in routine pediatric neuroimaging, including very short acquisition times without anesthesiology support, sufficient diagnostic interpretability with minimal need for additional imaging, and a reduction in CT utilization compared with a historical control cohort. Importantly, our cohort reflects real-world clinical use across a broad spectrum of indications rather than focusing on a single disease entity.

### 4.1. Adoption and Expansion of UF-MRI in Clinical Practice

Since its introduction in the early 2000s, the primary indication for ultrafast MRI has been the assessment of cerebrospinal fluid (CSF) spaces, particularly in children with (shunted) hydrocephalus. This is plausible given the high intrinsic contrast between CSF and brain parenchyma on heavily T2-weighted sequences. Several studies have demonstrated the reliability of UF-MRI for evaluating CSF spaces [9,11,12,13,15,28,29]. Consistent with this, hydrocephalus remained the most frequent diagnosis in our cohort, accounting for more than half of all examinations.

Beyond hydrocephalus, trauma and subdural hematomas, including abusive head trauma, were among the most common indications in our series. This reflects the evolution of the literature, in which UF-MRI has increasingly been evaluated in emergency settings, particularly for head trauma, since around 2015 [16,17,18,19,20,21,22,23,24,25,26,30,31,32]. Similarly to our study, most published series are retrospective, although a limited number of prospective investigations have been reported [16,17,21,24].

The diagnostic performance of UF-MRI in TBI appears context-dependent. Lindberg et al. [21] reported high sensitivity and specificity for clinically relevant TBI, and Sheridan et al. [24] demonstrated 100% sensitivity for injuries requiring acute clinical management. In contrast, Kralik et al. [17] reported limited sensitivity for subtle intracranial traumatic findings, with small subarachnoid and subdural hemorrhages missed in a substantial proportion of cases compared with conventional MRI. This highlights an important limitation: while UF-MRI appears reliable for detecting clinically relevant pathology, subtle traumatic findings of limited immediate clinical consequence may be overlooked. However, in the specific context of suspected abusive head trauma, such subtle findings may carry forensic relevance, which must be acknowledged as a limitation of ultrafast protocols.

A relevant proportion of examinations (11.7%) in our cohort were performed in emergency contexts such as headache, nausea/vomiting, or first epileptic seizure. This is consistent with previous reports. Ha et al. [33] demonstrated sufficient image quality of UF-MRI protocols for the evaluation of headache, congenital anomalies, and syncope. Luque et al. [18] showed that UF-MRI in children presenting with recurrent headache was clinically valuable, particularly for detecting sinusitis, and was associated with high levels of patient and family satisfaction during emergency department encounters. In our cohort, UF-MRI similarly contributed to the detection of clinically relevant intracranial pathology, including space-occupying lesions.

### 4.2. Workflow and Practical Considerations

Performing elective brain MRI under general anesthesia in pediatric patients, particularly infants and toddlers, entails considerable logistical complexity. The workflow requires extensive interdisciplinary coordination, including confirmation of indication, formal imaging requests, and allocation of limited anesthesia-supported MRI slots. The introduction of UF-MRI has substantially streamlined this process for outpatients. The workflows for conventional elective MRI and outpatient UF-MRI are illustrated in Supplementary Material S2.

Across published series, UF-MRI is consistently associated with very low or absent sedation rates [17,18,21,28,29,34], consistent with our findings and highlighting one of the key clinical advantages of ultrafast protocols. The need for sedation in preschool-aged children is largely driven by the examination duration: longer acquisitions are less well-tolerated and more susceptible to motion artifacts, often rendering images non-diagnostic.

Reported acquisition times for UF-MRI typically range from 1 to 5 min, depending on protocol complexity. The shortest protocols described by O’Neill et al. [12] achieved acquisition times of 12 s at 3 T and 19 s at 1.5 T, although these consisted of a single axial T2-HASTE sequence. In our cohort, individual sequences lasted 22–27 s, and approximately three-quarters of examinations were completed in under two minutes. Although total examination time inevitably exceeds pure acquisition time due to preparation and positioning, the entire workflow from patient entry to exit could be completed in approximately five minutes, comparable to real-world reports from other centers.

Our standardized protocol, based on multiplanar T2-HASTE sequences, offers robustness, high throughput, and ease of implementation in routine clinical practice, particularly in time-critical settings. However, studies using broader ultrafast protocols incorporating T1-weighted imaging, FLAIR, DWI, and T2* [21,26,33] have shown that expanded protocols with acquisition times as short as 1 min 11 s [33] can enhance the diagnostic scope, especially for non-hydrocephalus indications. These findings support a modular strategy, whereby a rapid core protocol is supplemented with targeted sequences such as FLAIR for screening, DWI for ischemia, and T2*/SWI for hemorrhage when clinically indicated.

We deliberately focused on children up to six years of age, as this group benefits the most from ultrafast MRI owing to limited cooperation and the frequent need for sedation with conventional protocols. In this age range, the advantages of motion tolerance and sedation avoidance are particularly clinically relevant. Nonetheless, several studies have also included older children and adolescents [15,20,26,30,34], which is justified given their continued radiation sensitivity and the substantial proportion of older pediatric patients with neurodevelopmental conditions that similarly limit cooperation.

### 4.3. Motion Artifacts and Diagnostic Adequacy

Motion artifacts were observed in approximately two-thirds of examinations, a higher proportion than reported in prior studies, which described moderate to severe motion in 24–27% of cases [12,16]. Despite this, diagnostic confidence remained high: although 40% of examinations exhibited pronounced artifacts, only one scan required repetition due to insufficient diagnostic adequacy. This likely reflects the robustness of the protocol, which is based on multiplanar T2-HASTE acquisitions and allows for the immediate repetition of sequences when motion is detected. Differences in reported artifact rates across studies are probably influenced by varying definitions; in our analysis, any visible motion artifact in at least one sequence was recorded, whereas other studies primarily considered artifacts that impaired diagnostic interpretability. Consistent with our findings, high diagnostic adequacy rates of 95–98% have been reported by others [12,15,16].

While UF-MRI proved sufficient for most clinical decisions, additional conventional MRI was mainly required for surgical planning or for the evaluation of epileptogenic foci. Lai et al. [35] demonstrated the feasibility of rapid MRI for neuronavigation in hydrocephalus without prolonging operative time, yet it remains unlikely that ultrafast protocols can fully replace high-resolution planning sequences for tumor surgery or comprehensive epilepsy imaging. Importantly, data on the impact of UF-MRI on concrete clinical and surgical decision-making remain limited in the literature, underscoring the added value of our real-world analysis

### 4.4. Impact on Imaging Utilization and CT Reduction

Following the implementation of UF-MRI, neurocranial MRI utilization in children younger than six years increased by more than 40% compared with the preceding 5.5-year period. This increase closely matches the number of UF-MRI examinations, while the number of conventional anesthesia-supported MRI studies remained nearly unchanged, suggesting that UF-MRI primarily compensated for previously unmet imaging demand rather than replacing conventional MRI. This interpretation is consistent with the persistently long waiting times for elective MRI under general anesthesia in routine practice.

Nevertheless, alternative explanations must be considered. Overall imaging demand has increased over time, and UF-MRI may have, in some cases, replaced examinations that would previously have been managed with CT, ultrasound, or clinical follow-up alone. While ultrasound utilization could not be reliably quantified, we were able to demonstrate a 41% reduction in CT examinations after UF-MRI implementation.

This finding is in line with previous reports. Iskandar proposed that UF-MRI could replace CT for shunted hydrocephalus follow-up as early as 2004 [9]. Niederhauser et al. [28] and Robson et al. [13] showed that UF-MRI became the predominant imaging modality for hydrocephalus and progressively replaced CT. In the context of suspected abusive head trauma, Berger et al. [16] suggested that UF-MRI-based screening could potentially reduce head CT use by more than 90%, and Franklin et al. [31] further reported that combining UF-MRI with skull radiography may substantially reduce the need for CT. Collectively, these data support the notion that UF-MRI can meaningfully contribute to reducing CT exposure in pediatric neuroimaging.

### 4.5. Limitations

This study has several limitations, including its retrospective single-center design and the absence of a direct prospective comparison with conventional MRI or CT protocols. Furthermore, image quality assessment was based on clinical usability rather than standardized quantitative metrics, which may limit direct comparability with other studies.

The observed reduction in CT utilization may have been influenced by changes in referral patterns, patient volume, or institutional imaging practices over time. Denominator data such as the number of CT examinations per 1000 neurosurgical encounters in this age group were not consistently available, and a relevant proportion of patients may have been coded under traumatology or general pediatrics rather than neurosurgery, which limits the ability to establish a causal relationship. Nevertheless, no major changes in institutional referral structures, catchment areas, or overall patient volume occurred during the study period, supporting UF-MRI implementation as a relevant contributing factor to the observed reduction in CT use.

In addition, workflow efficiency and clinical decision-making are influenced by institutional structures and local expertise, which may affect the generalizability of our findings to centers with different logistical or technical conditions, including MRI availability.

## 5. Conclusions

Ultrafast cranial MRI can be safely and effectively implemented for selected neuroimaging indications in children younger than six years. In our cohort, UF-MRI provided clinically sufficient information in the vast majority of examinations while reducing scan times, exposure to anesthesia, and ionizing radiation. Beyond its diagnostic value, UF-MRI streamlined clinical workflows and supported timely clinical decision-making. These findings support the integration of UF-MRI as a first-choice neuroimaging modality within the pediatric population for selected indications and highlight its potential to support patient-centered, resource-efficient care.

## Figures and Tables

**Table 1:** Patient demographics and examination characteristics.

Sex		N	
	Female	85	(42.9%)
	Male	113	(57.1%)
**Age at the time of MRI**			
	Neonates and infants (0–1 year)	109	(27.0%)
	Toddlers (1–3 years)	186	(46.0%)
	Preschoolers (4–6 years)	109	(27.0%)
**Clinical setting**			
	Inpatients	215	(53.2%)
	Outpatients	189	(46.8%)
**Clinical context**			
	Acutely symptomatic	175	(43.3%)
	Scheduled follow-up	209	(51.7%)
	Elective baseline MRI	20	(5.0%)
**Underlying diagnosis**			
	Posthemorrhagic hydrocephalus	91	(22.5%)
	Hydrocephalus of other origin	119	(29.5%)
	Traumatic brain injury	59	(14.6%)
	Subdural hematoma/hygroma	49	(12.1%)
	Intracranial cysts	14	(3.5%)
	Other diagnoses	72	(17.8%)
**Imaging indication**	(multiple choices possible)		
	Ventricular width	228	(56.4%)
	Dynamics of subdural hematoma/hygroma	92	(22.8%)
	Trauma sequelae	47	(11.6%)
	Work-up for epileptic seizures	32	(7.9%)
	Dynamics of cysts	20	(5.0%)
	Work-up for headache/vomiting	15	(3.7%)
	Others	50	(12.4%)

## Data Availability

The original contributions presented in this study are included in the article/Supplementary Material. Further inquiries can be directed to the corresponding author(s).

## Supplementary Materials

The following supporting information can be downloaded at https://www.mdpi.com/article/10.3390/jcm15031242/s1, Supplementary Table S1: Clinical consequences of UF-MRI according to underlying diagnosis, Supplementary Material S2: Workflow Conventional MRI vs. UF-MRI.

## Abbreviations

The following abbreviations are used in this manuscript:

UF-MRI	ultrafast cranial magnetic resonance tomography
cCT	cranial computed tomography
cMRI	cranial magnetic resonance tomography
CSF	cerebrospinal fluid
TBI	traumatic brain injury
