# Supplementary material for: Clinical Impact of Ultrafast Cranial MRI Implementation in Children Under Six Years of Age
Source: J Clin Med. 2026 Feb 4;15(3):1242. doi: 10.3390/jcm15031242 (PMC12898367; doi:10.3390/jcm15031242)
Supplement: Supplementary file 1 [file jcm-15-01242-s001.zip › Supplementary Table S1 Clinical consequences of UF-MRI according to underlying diagnosis.pdf]

Supplementary Table S1

Clinical consequences of UF-MRI according to underlying diagnosis

**Diagnosis**	**No specific measures n (%)**	**Early follow-up n (%)**	**Valve pressure reduction n (%)**	**Valve pressure increase n (%)**	**Surgery n (%)**	**Other measures n (%)**	**Total (N)**
Hydrocephalus after IVH	39 (41.9)	10 (10.8)	14 (15.1)	13 (14.0)	11 (11.8)	6 (6.5)	93
Hydrocephalus of other origin	51 (41.8)	33 (27.0)	12 (9.8)	4 (3.3)	14 (11.5)	8 (6.6)	122
Subdural haematoma / hygroma*	18 (36.7)	26 (53.1)	4 (8.2)	0 (0)	1 (2.0)	0 (0)	49
Trauma	43 (72.9)	15 (25.4)	0 (0)	0 (0)	1 (1.7)	0 (0)	59
Intracranial cysts	12 (85.7)	1 (7.1)	0 (0)	0 (0)	1 (7.1)	0 (0)	14
Other diagnoses	57 (78.1)	7 (9.6)	0 (0)	0 (0)	3(4.1)	6(8.2)	73

Percentages are calculated per diagnostic category.

IVH intraventricular haemorrhage

* incl. abusive head trauma
