# Supplementary figures and images for: Clinical Impact of Ultrafast Cranial MRI Implementation in Children Under Six Years of Age
Source: J Clin Med. 2026 Feb 4;15(3):1242. doi: 10.3390/jcm15031242 (PMC12898367; doi:10.3390/jcm15031242)

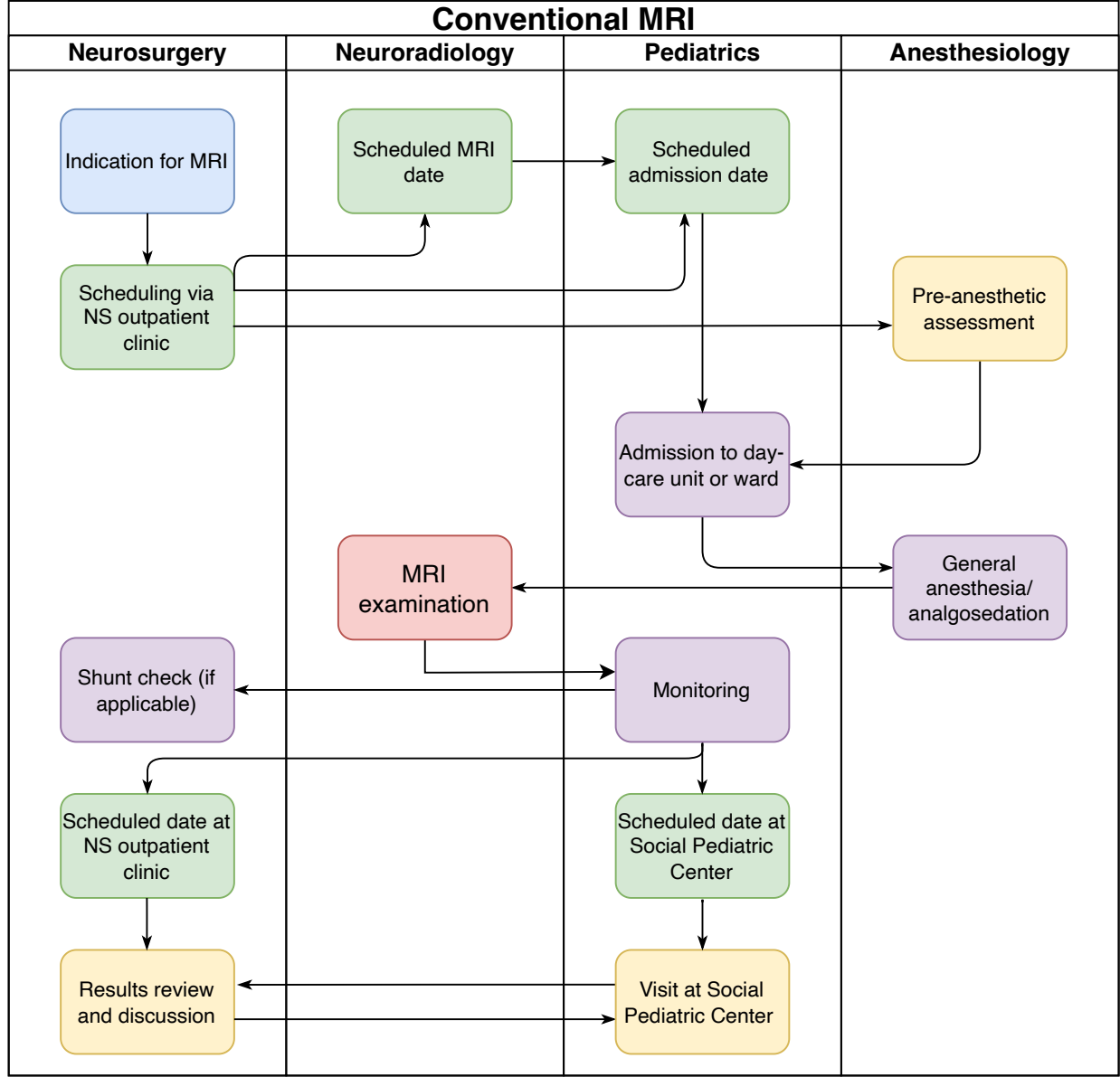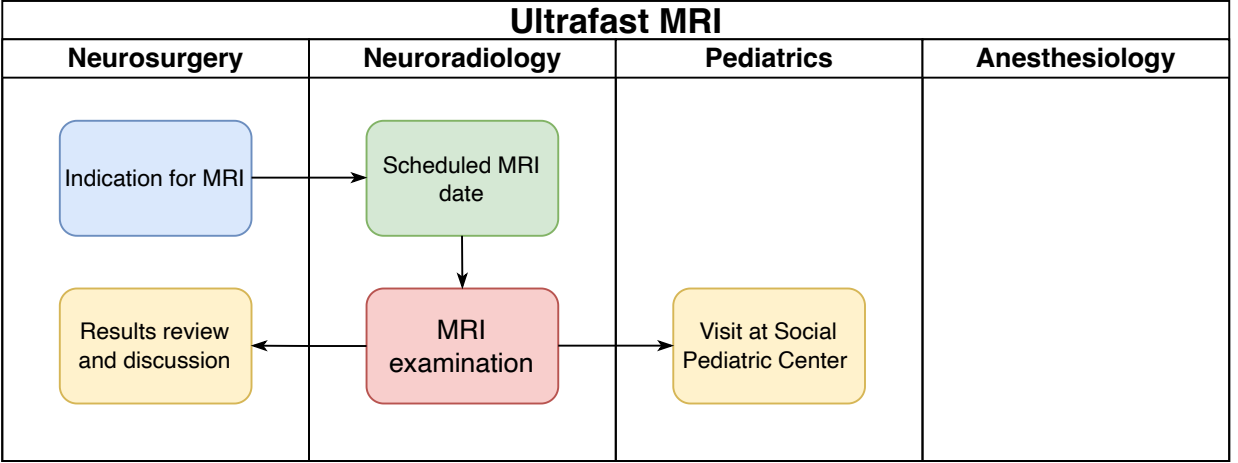

Supplement: Supplementary file 1 [file jcm-15-01242-s001.zip › Supplementary Material S2 Workflow Conventional MRI vs UF-MRI.pdf]
